# Supplementary figures and images for: Tensions on the actin cytoskeleton and apical cell junctions in the C. elegans spermatheca are influenced by spermathecal anatomy, ovulation state and activation of myosin
Source: Front Cell Dev Biol. 2024 Oct 15;12:1490803. doi: 10.3389/fcell.2024.1490803 (PMC11518831; doi:10.3389/fcell.2024.1490803)

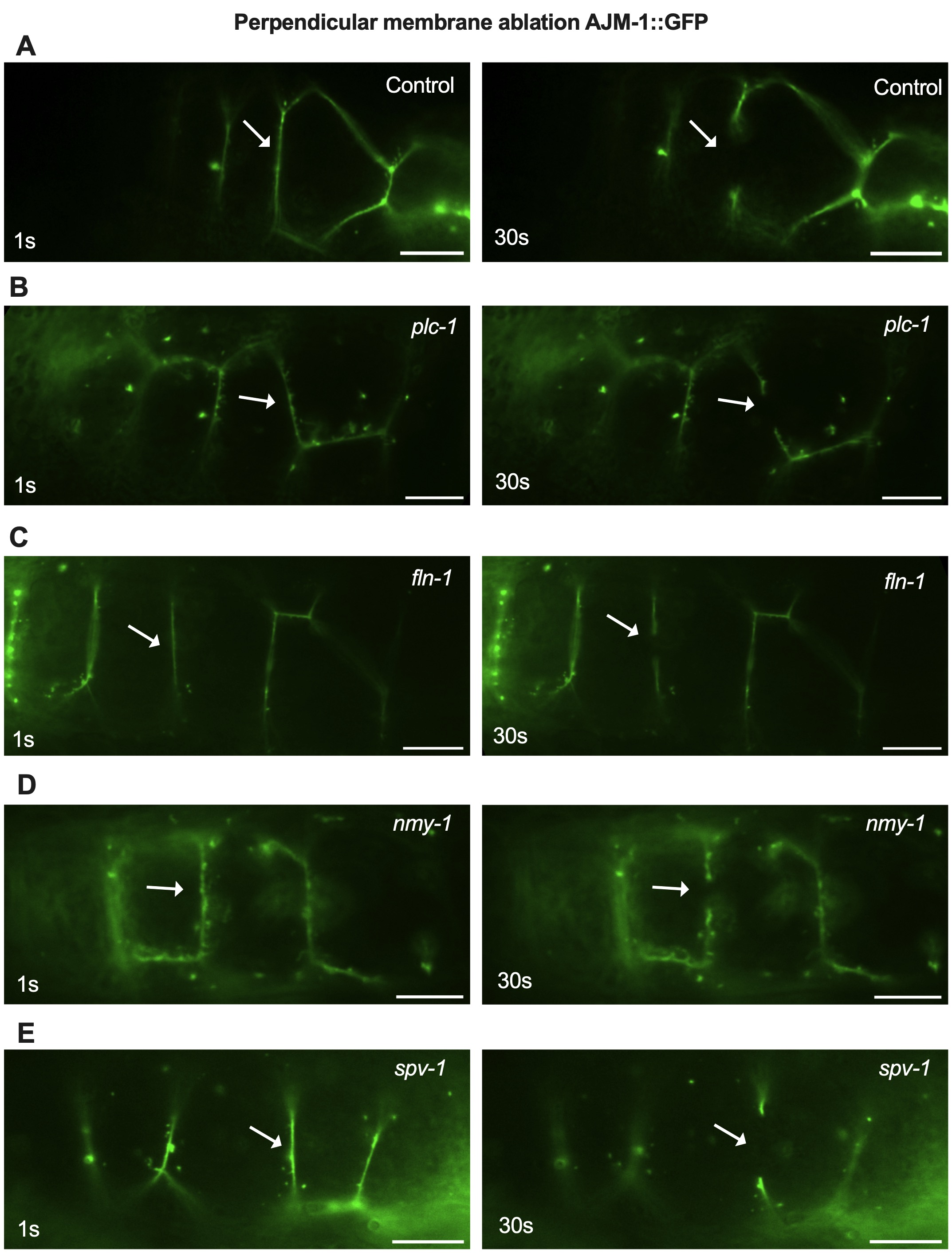

Supplement: Supplementary file 2 [file Image3.JPEG]

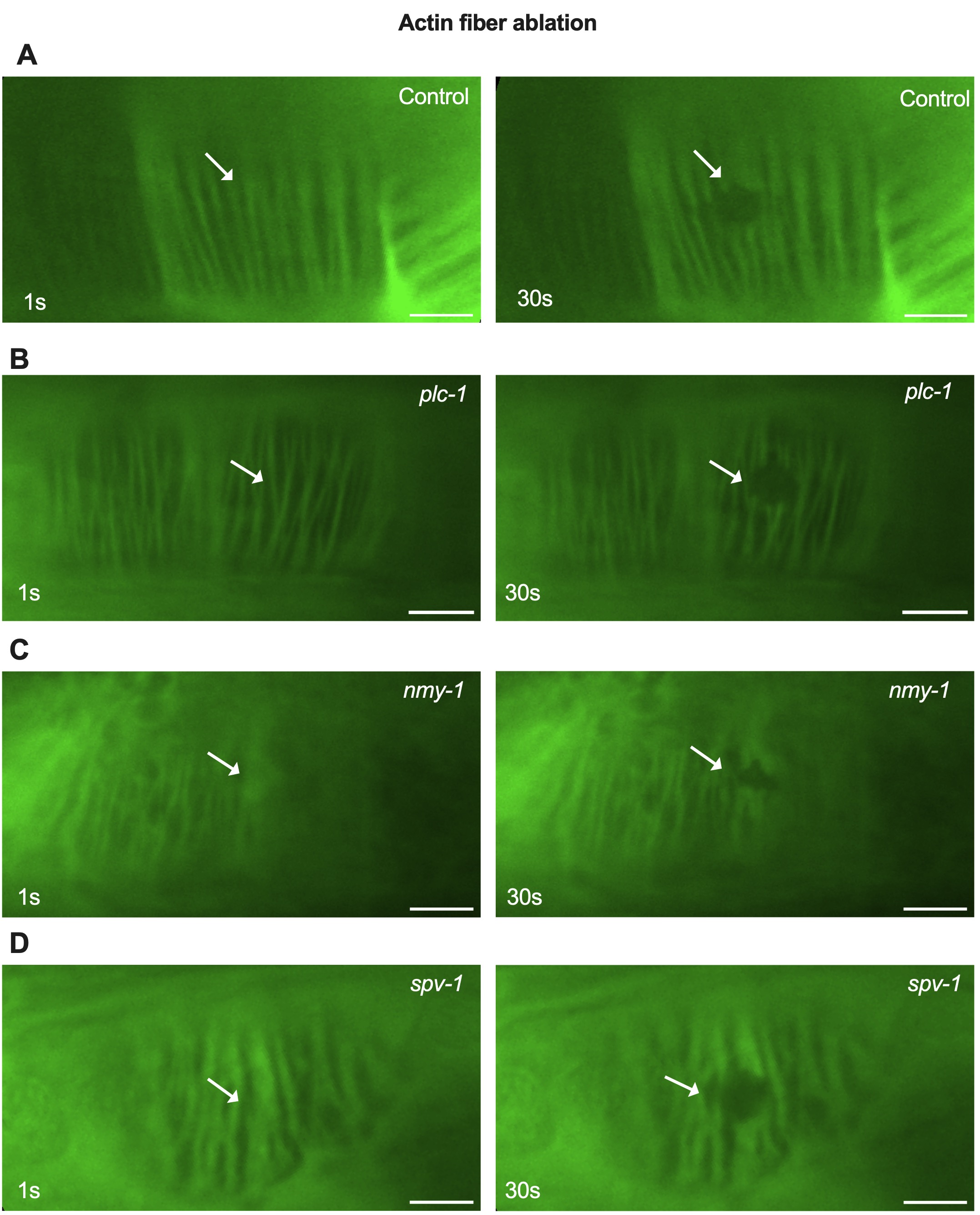

Supplement: Supplementary file 4 [file Image1.JPEG]

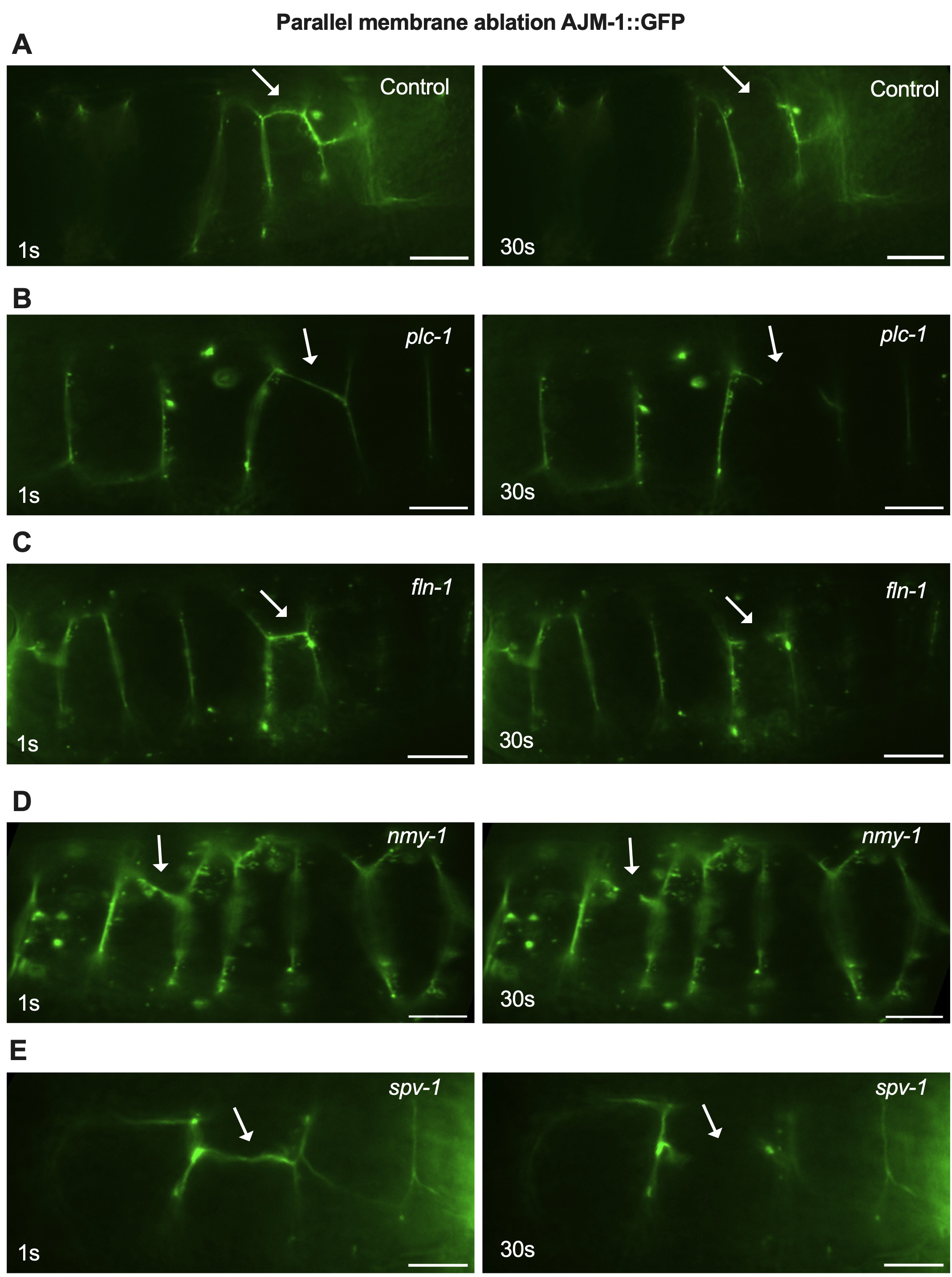

Supplement: Supplementary file 5 [file Image4.JPEG]

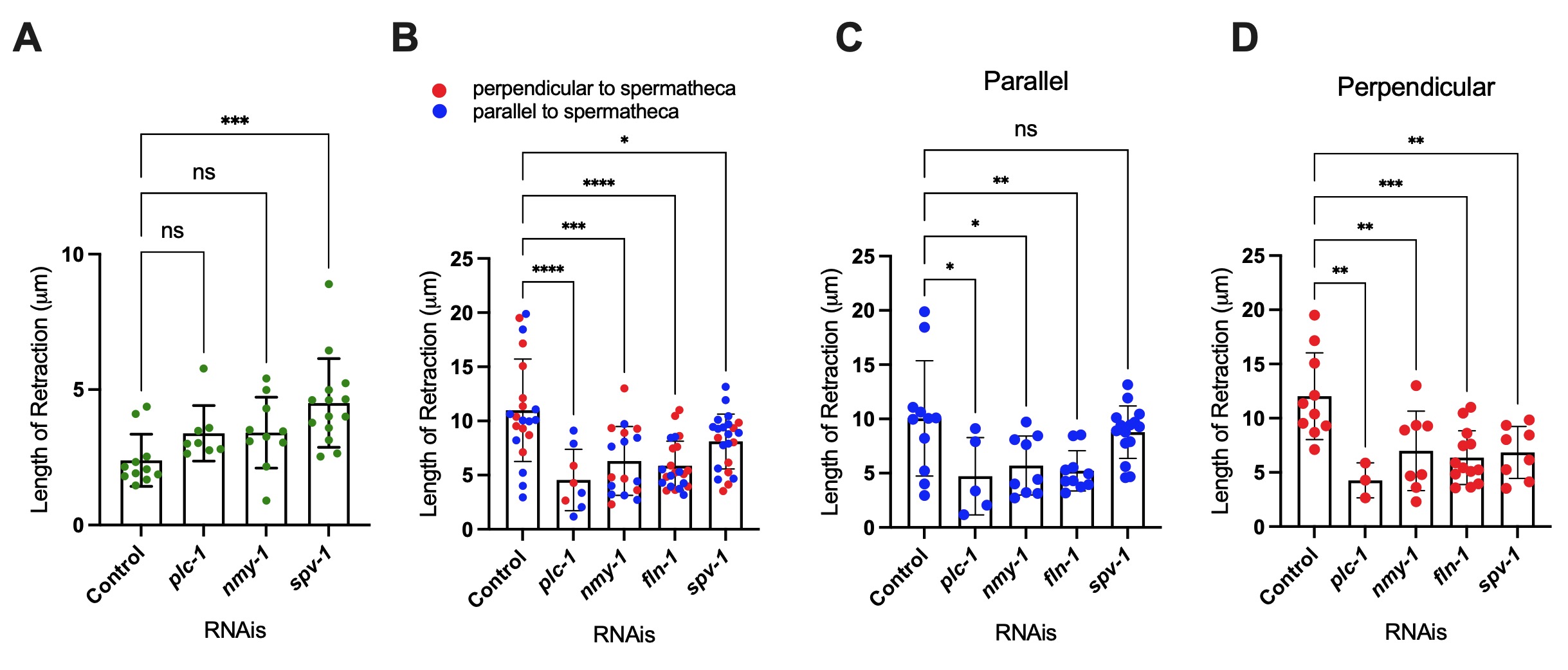

Supplement: Supplementary file 6 [file Image2.JPEG]
